# Supplementary material for: Simultaneous Onset of Haematological Malignancy and COVID: An Epicovideha Survey
Source: Cancers (Basel). 2022 Nov 10;14(22):5530. doi: 10.3390/cancers14225530 (PMC9688278; doi:10.3390/cancers14225530)
Supplement: Supplementary file 1 [file cancers-14-05530-s001.zip › cancers-1993485-supplementary.pdf]

Simultaneous onset of haematological malignancy and COVID: An EPICOVIDEHA survey

Supplementary tables

Table S1: COVID-19 directed antivirals

	n	%
**Casiririmivab + Imdevimab**	14	3.1%
**Remdesivir**	12	2.7%
**Casiririmivab + Imdevimab, Corticosteroids**	8	1.8%
**Remdesivir, Corticosteroids**	7	1.6%
**Sotrovimab, Corticosteroids**	5	1.1%
**Remdesivir, Casiririmivab + Imdevimab**	4	0.9%
**Remdesivir, Convalescent plasma, Corticosteroids**	3	0.7%
**NOS monoclonal antibodies, Corticosteroids**	2	0.4%
**Remdesivir, Convalescent plasma**	2	0.4%
**Remdesivir, Sotrovimab**	2	0.4%
**Sotrovimab**	2	0.4%
**Molnupiravir**	1	0.2%
**Molnupiravir, Corticosteroids**	1	0.2%
**NOS monoclonal antibodies**	1	0.2%
**Remdesivir, Casiririmivab + Imdevimab, Corticosteroids**	1	0.2%
**Remdesivir, NOS monoclonal antibodies**	1	0.2%
**Remdesivir, Sotrovimab, Corticosteroids**	1	0.2%

Table S2: HM treatments before and after COVID-19 diagnosis

	n	%
**Last malignancy treatment before COVID-19 diagnosis**	227	50.4%
Conventional chemotherapy	79	34.8%
Conventional chemotherapy + Hypomethylating agents	1	0.4%
Conventional chemotherapy + IMiD	13	5.7%
Conventional chemotherapy + Targeted therapy	8	3.5%
Growth factors	1	0.4%
Hypomethylating agents	10	4.4%
Hypomethylating agents + Targeted therapy	10	4.4%
IMiD	1	0.4%
Immunochemotherapy	52	22.9%
Immunotherapy	4	1.8%
Palliative/supportive measures	32	14.1%
Targeted therapy	14	6.2%
Unknown	2	0.9%
**Malignancy treatment after COVID-19 diagnosis**	217	48.2%
Conventional chemotherapy	58	26.7%
Conventional chemotherapy + Hypomethylating agents	1	0.5%
Conventional chemotherapy + IMiD	7	3.2%
Conventional chemotherapy + IMiD + Radiotherapy	1	0.5%
Conventional chemotherapy + Targeted therapy	4	1.8%
Growth factors	1	0.5%
Hypomethylating agents	7	3.2%
Hypomethylating agents + Targeted therapy	6	2.8%
IMiD	1	0.5%
Immunochemotherapy	60	27.6%
Immunochemotherapy + IMiD	1	0.5%
Immunochemotherapy + Radiotherapy	1	0.5%
Immunotherapy	5	2.3%
Immunotherapy + IMiD	1	0.5%
Palliative/supportive measures	32	14.7%
Radiotherapy	1	0.5%
Targeted therapy	17	7.8%
Unknown	13	6.0%

COVID-19, coronavirus disease 2019; IMiD, immunomodulatory imide drugs

Table S3. 30-day mortality according to HM treatment start

Time HM treatment	Total	Deceased at 30 days (%)
No treatment	107	45 (42.1%)
Only after COVID-19	116	14 (12.1%)
At least before COVID-19 *	108	52 (48.1%)
Before and after COVID-19	101	19 (18.8%)
Only before COVID-19	18	9 (50.0%)

COVID-19, coronavirus disease 2019; HM, haematological malignancy

* "At least before COVID-19" stands for patients with known HM treatment before COVID-19, but unknown after COVID-19

Table S4. Univariable and multivariable analysis for risk factors for mortality (AML patients)

	Univariable				Multivariable			
	p value	HR	95% CI Lower	95% CI Upper	p value	HR	95% CI Lower	95% CI Upper
**Sex**								
Female	-	-	-	-				
Male	0.737	0.922	0.573	1.483				
**Age**	0.002	1.026	1.009	1.043	0.022	1.021	1.003	1.039
**COVID-19 infection severity°**								
Asymptomatic	-	-	-	-	-	-	-	-
Mild infection	0.949	0.966	0.335	2.783	0.991	0.994	0.344	2.868
Severe infection	0.591	1.222	0.588	2.541	0.941	1.029	0.489	2.164
Critical infection	<0.001	3.161	1.595	6.263	0.005	2.716	1.363	5.412
**Comorbidities at COVID-19 onset**								
No comorbidities	-	-	-	-				
1 comorbidity	0.826	0.938	0.531	1.657				
2 comorbidities	0.344	1.367	0.716	2.608				
3 or more comorbidities	0.518	1.322	0.567	3.083				
**Season COVID-19 diagnosis**								
March 2020 - September 2020	-	-	-	-	-	-	-	-
October 2020 - February 2021	0.133	0.669	0.396	1.130	0.897	1.038	0.590	1.825
March 2021 - November 2021	0.229	0.601	0.262	1.379	0.856	0.916	0.355	2.364
December 2021 - February 2022	0.029	0.395	0.171	0.911	0.779	0.847	0.267	2.690
**Malignancy treatment?**								
No	-	-	-	-				
Before COVID-19 diagnosis	0.002	0.375	0.200	0.705				
After COVID-19 diagnosis	<0.001	0.225	0.102	0.494				
**Chemotherapy induced neutropenia**								
No	-	-	-	-	-	-	-	-
Yes	0.292	0.751	0.441	1.279	0.694	0.895	0.515	1.556
No treatment administered	0.007	2.559	1.287	5.089	0.008	2.560	1.278	5.129
**Secondary infection after COVID-19**	**0.552**	**1.184**	**0.679**	**2.066**				
**COVID-19 treatment**								
No specific treatment reported	-	-	-	-	-	-	-	-
Antivirals + monoclonal antibodies ± corticosteroids ± plasma	0.972	0.000	0.000	.	0.977	0.000	0.000	.
Antivirals ± corticosteroids ± plasma	0.540	0.800	0.391	1.635	0.953	0.978	0.465	2.056
Corticosteroids	0.970	0.986	0.484	2.011	0.802	1.097	0.532	2.266

Table S4. Univariable and multivariable analysis for risk factors for mortality (AML patients)

	Univariable				Multivariable			
	p value	HR	95% CI		p value	HR	95% CI	
			Lower	Upper			Lower	Upper
Monoclonal antibodies ± plasma ± corticosteroids	0.071	0.271	0.066	1.119	0.221	0.394	0.088	1.754
Plasma ± corticosteroids	0.289	0.343	0.047	2.483	0.164	0.237	0.031	1.796

AML, acute myeloid leukaemia; CI, confidence interval; COVID-19, coronavirus disease 2019; HR, hazard ratio

°asymptomatic: no clinical signs or symptoms; mild: non-pneumonia and mild pneumonia; severe: dyspnea, respiratory frequency ≥ 30 breaths per min, $SpO_2 \leq 93\%$, $PaO_2/FiO_2 < 300$, or lung infiltrates > 50%); critical: patients admitted in intensive care for respiratory failure, septic shock, or multiple organ dysfunction or failure

**Table S5.** Univariable and multivariable analysis for risk factors for mortality (Lymphoma patients)								
	**Univariable**				**Multivariable**			
	**p value**	**HR**	**95% CI Lower**	**95% CI Upper**	**p value**	**HR**	**95% CI Lower**	**95% CI Upper**
**Sex**								
Female	-	-	-	-				
Male	0.803	1.076	0.603	1.920				
**Age**	<0.001	1.055	1.031	1.080	<0.001	1.075	1.045	1.105
**Type of lymphoma**								
Indolent	-	-	-	-				
Aggressive	0.969	1.012	0.540	1.898				
**COVID-19 infection severity°**								
Asymptomatic	-	-	-	-	-	-	-	-
Mild infection	0.860	1.138	0.272	4.761	0.359	2.038	0.445	9.334
Severe infection	0.063	2.580	0.951	6.996	0.337	1.657	0.591	4.646
Critical infection	<0.001	7.641	2.887	20.226	<0.001	6.461	2.354	17.734
**Comorbidities at COVID-19 onset**								
No comorbidities	-	-	-	-	-	-	-	-
1 comorbidity	0.095	1.959	0.889	4.315	0.462	1.362	0.598	3.101
2 comorbidities	<0.001	3.912	1.793	8.535	0.135	2.227	0.780	6.357
3 or more comorbidities	0.374	1.538	0.596	3.969	0.907	0.942	0.349	2.547
**Season COVID-19 diagnosis**								
March 2020 - September 2020	-	-	-	-	-	-	-	-
October 2020 - February 2021	0.070	0.540	0.278	1.051	0.018	0.398	0.185	0.856
March 2021 - November 2021	0.387	0.694	0.303	1.588	0.025	0.355	0.143	0.880
December 2021 - February 2022	0.025	0.289	0.097	0.858	0.047	0.319	0.103	0.984
**Malignancy treatment start**								
No	-	-	-	-				
Before COVID-19 diagnosis	0.106	0.589	0.309	1.120				
After COVID-19 diagnosis	<0.001	0.092	0.030	0.281				
**Chemotherapy induced neutropenia**								
No	-	-	-	-	-	-	-	-
Yes	0.009	2.554	1.270	5.140	0.002	3.333	1.572	7.067
**No treatment administered**	<0.001	4.408	2.052	9.466	<0.001	5.462	2.332	12.791
**Secondary infection after COVID-19**	0.286	0.648	0.292	1.438				
**COVID-19 treatment**								
No specific treatment reported	-	-	-	-				

**Table S5.** Univariable and multivariable analysis for risk factors for mortality (Lymphoma patients)								
	**Univariable**				**Multivariable**			
	**p value**	**HR**	**95% CI**		**p value**	**HR**	**95% CI**	
			**Lower**	**Upper**			**Lower**	**Upper**
Antivirals + monoclonal antibodies ± corticosteroids ± plasma	0.494	0.498	0.068	3.673				
Antivirals ± corticosteroids ± plasma	0.209	1.659	0.754	3.652				
Corticosteroids	0.152	1.735	0.817	3.688				
Monoclonal antibodies ± plasma ± corticosteroids	0.211	0.280	0.038	2.060				
Plasma ± corticosteroids	0.980	0.000	0.000	.				

CI, confidence interval; COVID-19, coronavirus disease 2019; HR, hazard ratio

asymptomatic: no clinical signs or symptoms; mild: non-pneumonia and mild pneumonia; severe: dyspnea, respiratory frequency ≥ 30 breaths per min, SpO2 ≤ 93%, PaO2/FiO2 < 300, or lung infiltrates > 50%); critical: patients admitted in intensive care for respiratory failure, septic shock, or multiple organ dysfunction or failure

Supplementary figures

Figure legends

Figure S1. 30-day survival probability of treated vs untreated patients according to HM

Figure S1 a. Acute lymphoid leukaemia
Figure S1 b. Acute myeloid leukaemia
Figure S1 c. Lymphoma
Figure S1 d. Chronic lymphoproliferative disorders
Figure S1 e. Multiple myeloma
Figure S1 f. Myelodysplastic syndrome + Myeloproliferative disorder

Figure S1.

Figure S1 a.

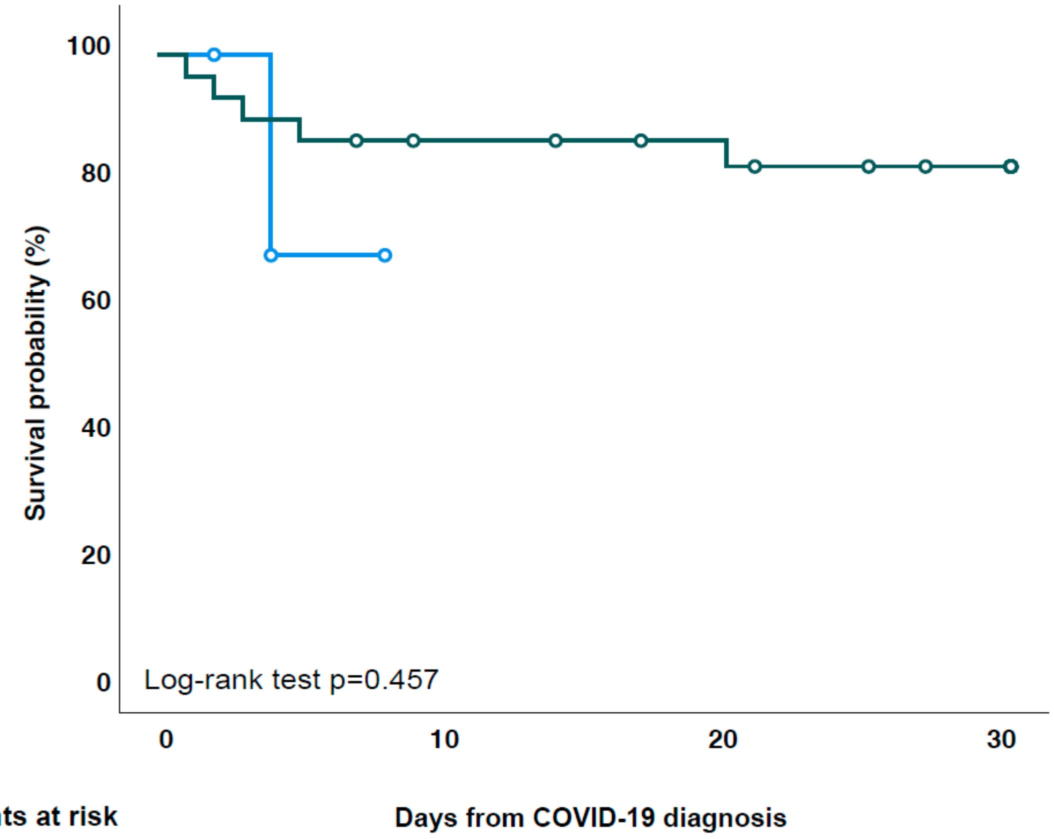

Number of patients at risk				
No HM treatment	4	0	0	0
HM treatment	28	22	20	16

Figure S1 b.

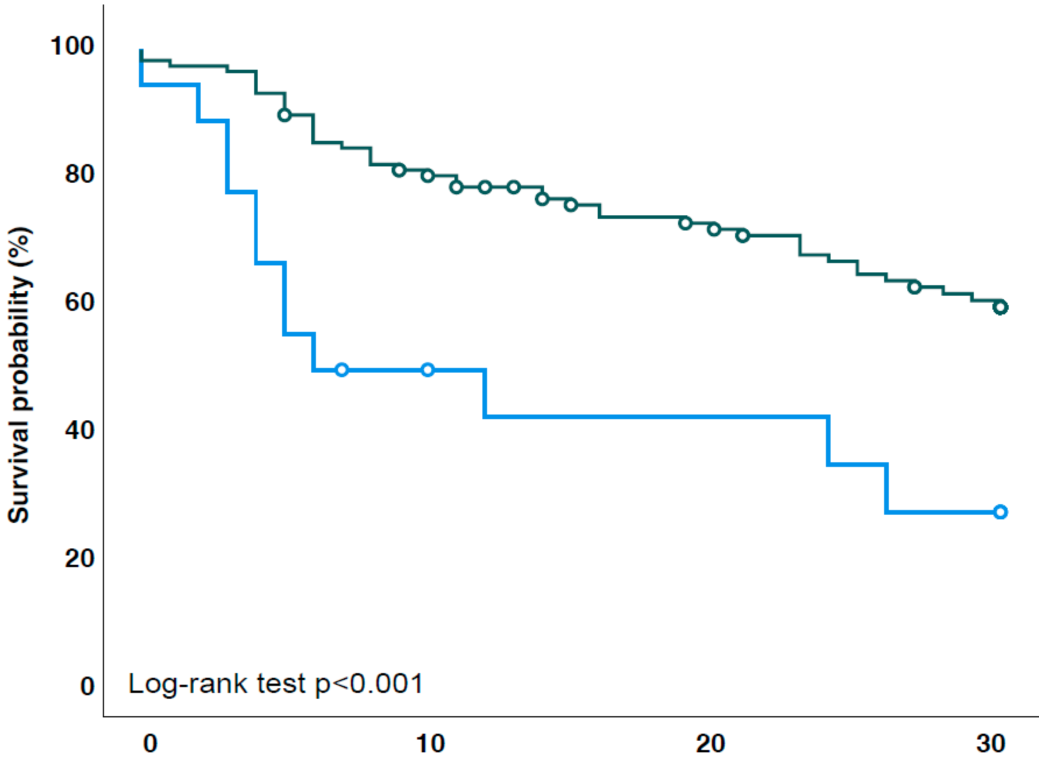

Number of patients at risk	Days from COVID-19 diagnosis			
No HM treatment	17	7	5	3
HM treatment	111	86	70	53

Figure S1 c.

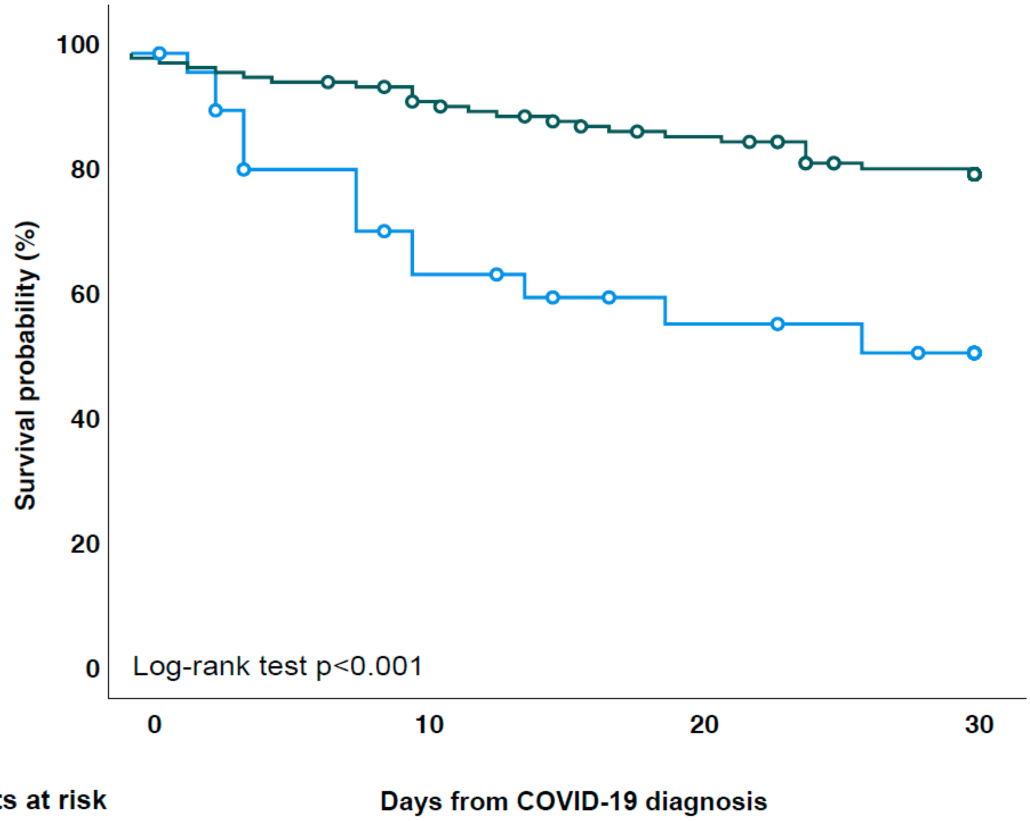

Number of patients at risk				
No HM treatment	32	19	12	9
HM treatment	123	114	98	85

Figure S1 d.

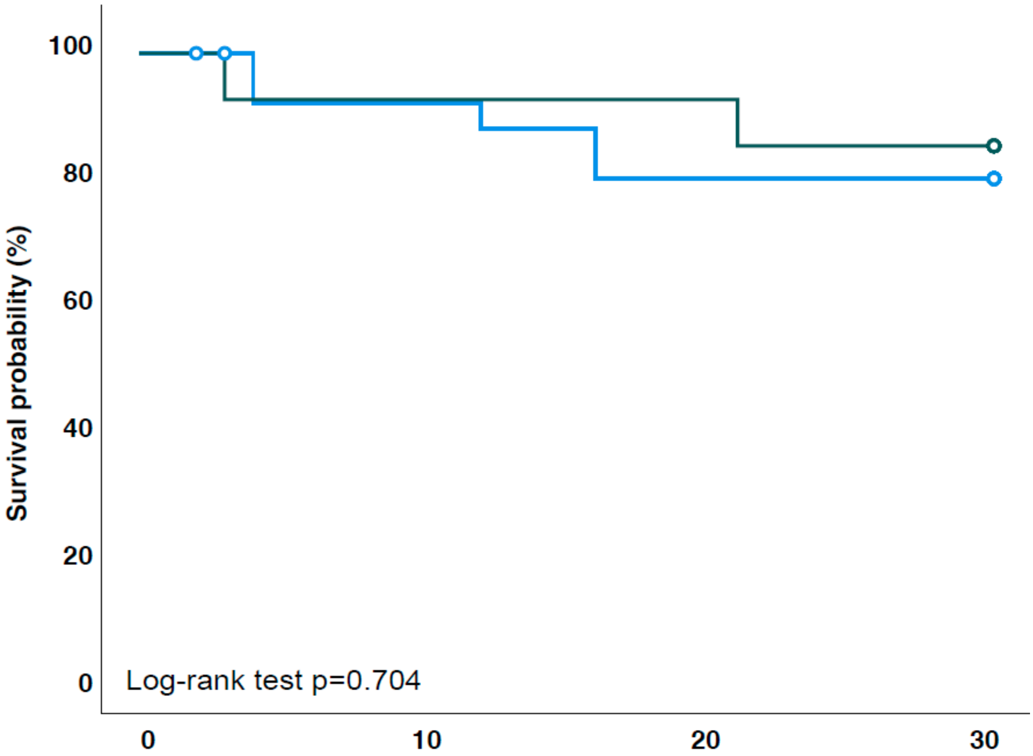

Number of patients at risk	Days from COVID-19 diagnosis			
No HM treatment	26	22	19	19
HM treatment	13	12	12	11

Figure S1 e.

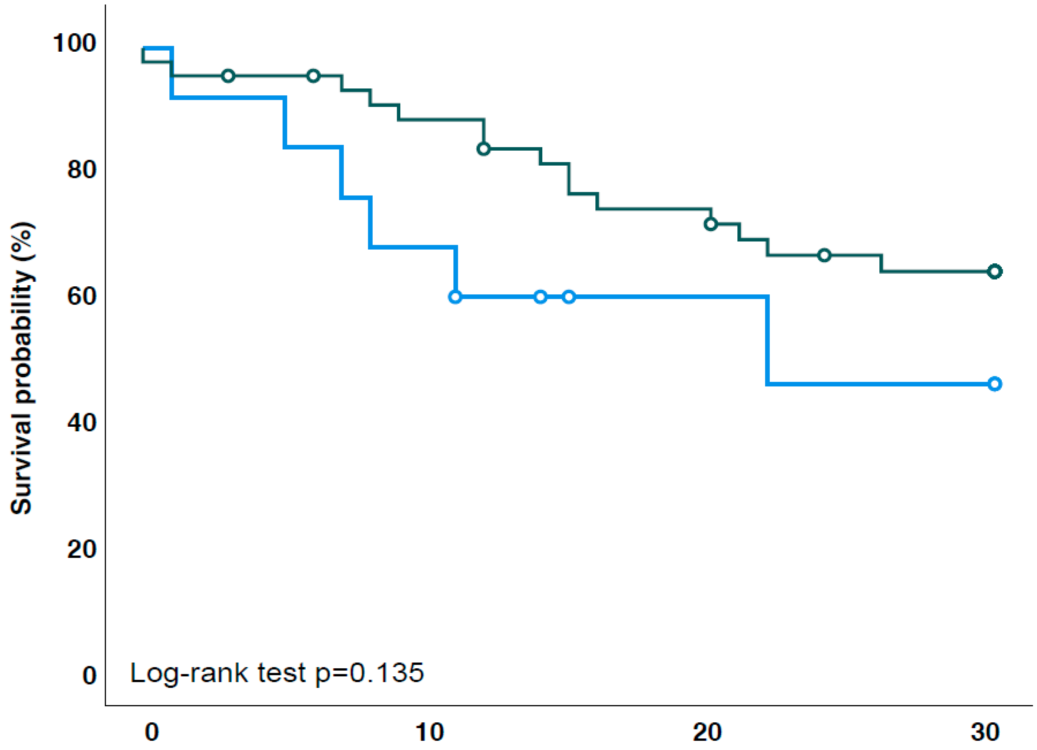

Number of patients at risk	Days from COVID-19 diagnosis			
No HM treatment	12	8	4	3
HM treatment	43	36	29	23

Figure S1 f.

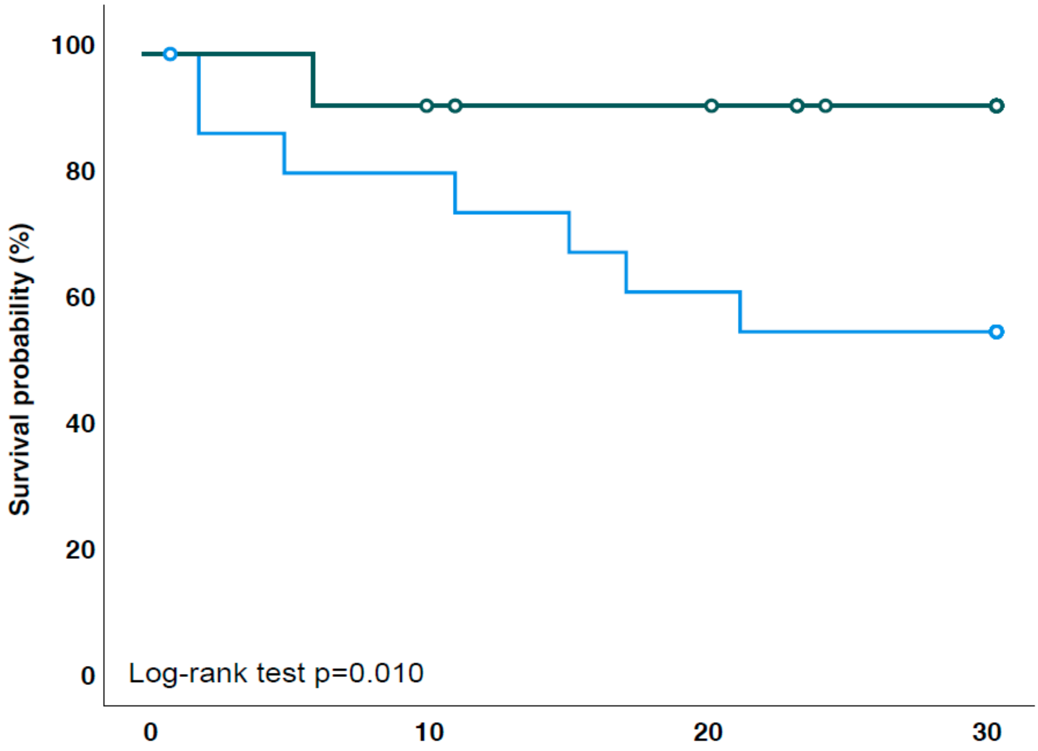

Number of patients at risk — **Days from COVID-19 diagnosis**

No HM treatment	16	12	9	8
HM treatment	23	21	18	13
